# A phase 3, randomized, double-blind, active-controlled clinical trial to compare BAT1806/BIIB800, a tocilizumab biosimilar, with tocilizumab reference product in participants with moderate-to-severe rheumatoid arthritis with inadequate response to methotrexate: treatment period 2 analysis (week 24 to week 48)

**DOI:** 10.1186/s13075-024-03375-w

**Published:** 2024-09-07

**Authors:** Xiaomei Leng, Piotr Leszczyński, Slawomir Jeka, Shengyun Liu, Huaxiang Liu, Malgorzata Miakisz, Jieruo Gu, Lali Kilasonia, Mykola Stanislavchuk, Xiaolei Yang, Yinbo Zhou, Qingfeng Dong, Marian Mitroiu, Janet Addison, Mourad F. Rezk, Xiaofeng Zeng

**Affiliations:** 1 Department of Rheumatology and Immunology, Chinese Academy of Medical Sciences & Peking Union Medical College Hospital, Beijing, China; 2 Department of Internal Medicine, Poznan University of Medical Sciences, Poznan, Poland; 3 Department of Rheumatology and Connective Tissue Diseases, University Hospital No 2, CM UMK, Bydgoszcz, Poland; 4 First Affiliated Hospital of Zhengzhou University, Zhengzhou, China; 5 Qilu Hospital of Shandong University, Jinan, China; 6 Twoja Przychodnia Centrum Medyczne, Nowa Sól, Poland; 7 The Third Affiliated Hospital of Sun Yat-sen University, Guangzhou, China; 8 Tbilisi Heart and Vascular Clinic Ltd, Tbilisi, Georgia; 9 National Pirogov Memorial Medical University, Vinnytsia, Ukraine; 10 Bio-Thera Solutions Ltd, Guangzhou, China; 11 Biogen International GmbH, Baar, Switzerland; 12 Biogen Idec Ltd, Maidenhead, UK

**Keywords:** Rheumatoid arthritis, Biosimilar pharmaceuticals, Tocilizumab, Treatment switching

## Abstract

**Background:**

Equivalent efficacy and comparable pharmacokinetic, immunogenicity, and safety profiles of the biosimilar BAT1806/BIIB800 and reference tocilizumab (TCZ) in participants with moderate-to-severe rheumatoid arthritis (RA) have been reported up to week 24 (treatment period [TP] 1) of the phase 3 study. Here we present results for TP2 (study weeks 24–48).

**Methods:**

In this phase 3, multicenter, multiregional, double-blind, active-controlled, equivalence study, participants with active RA despite methotrexate were randomized (1:1:2) to intravenous administration of 8 mg/kg TCZ every 4 weeks to week 48 (TCZ group), or TCZ to week 24 followed by BAT1806/BIIB800 to week 48 (TCZ to BAT1806/BIIB800 group), or BAT1806/BIIB800 to week 48 (BAT1806/BIIB800 group). Efficacy in TP2 was evaluated using American College of Rheumatology (ACR) response criteria (ACR20/50/70) and change from baseline in Disease Activity Score on 28 joints (DAS28). Pharmacokinetics (trough levels), safety, and immunogenicity were also evaluated.

**Results:**

Of 621 randomized participants, 577 (92.9%) completed TP1 and entered TP2 (TCZ: *N* = 145 [93.5%]; TCZ to BAT1806/BIIB800: *N* = 142 [92.2%]; BAT1806/BIIB800: *N* = 290 [92.9%]). Proportions of ACR20 responders were similar between treatment groups throughout TP2 (87.8%, 90.3%, and 90.4%, respectively, at week 48), as were proportions of ACR50 and ACR70 responders, and reduction in DAS28. Drug trough levels and antidrug antibody incidences were comparable between the treatment groups. Adverse events were balanced across the treatment groups and no fatal events were reported.

**Conclusion:**

In TP2, efficacy, safety, immunogenicity, and pharmacokinetic profiles were comparable between the TCZ, TCZ to BAT1806/BIIB800, and BAT1806/BIIB800 groups.

**Trial registration:**

NCT03830203 and EudraCT 2018-002202-31.

**Supplementary Information:**

The online version contains supplementary material available at 10.1186/s13075-024-03375-w.

## Background

Tocilizumab was the first humanized anti–interleukin-6 receptor (IL-6R) monoclonal antibody to be approved for the treatment of rheumatoid arthritis (RA). BAT1806/BIIB800 is an IL-6R–targeted, recombinant, humanized, monoclonal immunoglobulin G1 antibody developed in accordance with regulatory guidance as a biosimilar of the tocilizumab reference product (TCZ; Actemra™/RoActemra™) [1–3].

The development of biosimilars follows a stepwise approach to demonstrate similarity between the biosimilar and the reference product in terms of physicochemical characteristics, biological activity and pharmacokinetics (PK)/pharmacodynamics. For BAT1806/BIIB800, a phase 1 clinical study in healthy volunteers demonstrated equivalent PK and comparable safety and immunogenicity profiles following intravenous administration of a single dose of BAT1806/BIIB800 or TCZ [4]. A phase 3, multicenter, multiregional, randomized, double-blind, active-controlled equivalence study was conducted to demonstrate the clinical similarity of BAT1806/BIIB800 and TCZ administered intravenously in a population with moderate-to-severe RA with inadequate response to methotrexate (MTX). Findings from the initial 24 weeks (treatment period [TP] 1) of that trial demonstrated equivalent efficacy and similar safety, immunogenicity, and PK profiles of BAT1806/BIIB800 compared with TCZ at weeks 12 and 24 after initiation of study treatment [5].

Here the efficacy results for TP2 (i.e., week 24 to week 48), and safety, PK, and immunogenicity findings from week 24 to week 52 are reported.

## Methods

### Study design

This was a phase 3, multicenter, multiregional, randomized, double-blind, active-controlled clinical trial [5]. The study comprised a ≤ 28-day screening period, an initial 24-week period (TP1), and a subsequent 24-week period (TP2).

Eligible participants were randomized (1:1:2) using an interactive web-response system to receive TCZ up to week 48 (TCZ group), or TCZ up to week 24 followed by BAT1806/BIIB800 up to week 48 (TCZ→BAT1806/BIIB800 group), or BAT1806/BIIB800 up to week 48 (BAT1806/BIIB800 group). All study drugs were administered intravenously at a dose of 8 mg/kg once every 4 weeks. Randomization was stratified by geographical region and by previous use of biological disease-modifying antirheumatic drugs (DMARDs), or targeted synthetic DMARDs [5].

### Participants

All participants provided informed consent to participate, and the study was conducted in accordance with the Declaration of Helsinki and all relevant local regulations, in compliance with the International Council for Harmonisation Good Clinical Practice guidelines, and according to the appropriate regulatory requirements in the countries where the study was conducted.

Details of the study eligibility criteria are reported elsewhere [5]. In brief, eligible adults had an RA diagnosis of ≥ 6 months prior to screening, active disease (tender joint count ≥ 6 out of 68 joints and swollen joint count ≥ 6 out of 66 joints) and, at screening, a serum C-reactive protein (CRP) level greater than the upper limit of normal or erythrocyte sedimentation rate (ESR) ≥ 28 mm/h, despite receiving a stable dose of MTX (ranging between 10 and 25 mg/week). Prior treatment with more than two biological DMARDs or targeted synthetic DMARDs was not permitted, nor was treatment with any IL-6 inhibitor. Participants were expected to remain on their stable dose of MTX throughout the study.

### Endpoints

Efficacy in TP2 was assessed as the proportion of participants achieving ≥ 20% improvement over time (week 28 to week 48) in American College of Rheumatology (ACR) response criteria (ACR20), ≥50% improvement in ACR response criteria (ACR50), and ≥ 70% improvement in ACR response criteria (ACR70), and change over time (week 28 to week 48) from baseline (week 0) in Disease Activity Score on 28 joints using ESR (DAS28-ESR) and Disease Activity Score on 28 joints using CRP (DAS28-CRP).

PK in TP2 was assessed as tocilizumab serum trough concentration (C_trough_), measured at weeks 28, 36, 44, and 48, and at the end-of-study follow-up visit (i.e., week 52) or at participant withdrawal, whichever occurred first.

Safety, assessed throughout TP2 and up to the end-of-study follow-up visit at week 52, was determined by monitoring vital signs, physical examination, laboratory assessments (hematology, chemistry with lipids panel, and urinalysis), 12-lead electrocardiogram, and reporting of adverse events (AEs) including treatment-emergent AEs (TEAEs), serious TEAEs, study-drug-related TEAEs, and study-drug-related serious TEAEs. Immunogenicity was assessed by the presence of antidrug antibodies (ADAs), including neutralizing antibodies (nAbs), and ADA titers at weeks 28, 36, and 48, and at the end-of-study follow-up visit at week 52 or 8 weeks after the last dose of study drug, whichever occurred first.

### Statistical analysis

Efficacy analyses were performed by randomized treatment group sequence in participants receiving TCZ, TCZ→BAT1806/BIIB800, or BAT1806/BIIB800. The clinical efficacy endpoints (ACR20, ACR50, and ACR70, and change from baseline in DAS28-ESR and DAS28-CRP) for TP2 were analyzed on the full analysis set, which included all randomized participants who completed TP1 and entered TP2, using all observed outcome values. For DAS28 (ESR and CRP), least squares means for each group were estimated at each visit with a mixed model for repeated measures (MMRM) with the factors treatment arm, visit, randomized strata (region [Central Europe/Asia Pacific] and previous biologic or targeted synthetic DMARD use [Yes/No]), baseline, and treatment-by-visit interaction in the model, with and without missing data imputation, respectively. Missing data were imputed using baseline observation carried forward (BOCF) to reflect a nonresponder imputation. These MMRM analyses (for TP2) were not prespecified in the study statistical analysis plan, they were additionally requested by Regulatory Agencies during the scientific evaluation of the medicinal product.

Descriptive statistics of the observed clinical outcomes (ACR, ADAs, and intercurrent events) are provided.

Safety, immunogenicity, and PK endpoints were summarized descriptively. The safety set, as defined in the study statistical analysis plan, included all randomized participants who completed TP1, entered TP2, and who received at least one dose of study drug. All safety and immunogenicity endpoint analyses were performed on the safety set. Analysis of PK data was performed on the PK set, which included all randomized participants who had received at least one dose of study drug and had ≥ 1 evaluable PK assessment postbaseline.

All statistical analyses were conducted using SAS statistical software version 9.4 (SAS Institute Inc., Cary, NC, USA).

## Results

**Fig. 1:**
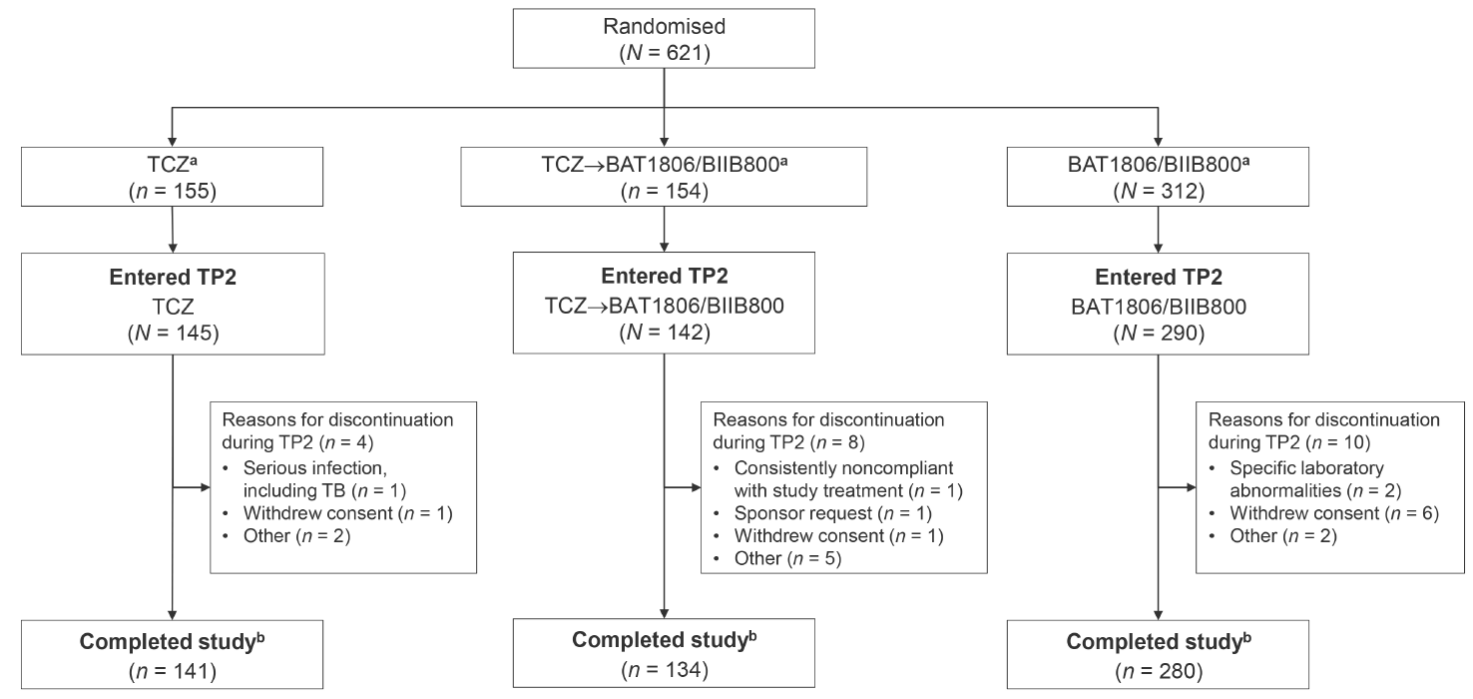
Trial disposition for TP2. ^a^ Eligible participants were randomized (1:1:2) to receive TCZ up to week 48 (TCZ), TCZ up to week 24 followed by BAT1806/BIIB800 up to week 48 (TCZ→BAT1806/BIIB800), or BAT1806/BIIB800 up to week 48 (BAT1806/BIIB800). ^b^ Participants were noted as having completed the study if they completed the follow-up visit, regardless of whether they completed TP2 (defined as completing week 48/EOS visit). *EOS* End of study, *TB* Tuberculosis, *TCZ* Tocilizumab reference product, *TP2* Treatment period 2

Between December 19, 2018 and January 5, 2021, 935 participants were screened and 621 eligible participants were randomized to study treatment (TCZ, *N* = 155; TCZ→BAT1806/BIIB800, *N* = 154; BAT1806/BIIB800, *N* = 312; Fig. 1). Of the 621 randomized participants, 577 (92.9%) completed TP1 and entered TP2 (*N* = 145 [93.5%], *N* = 142 [92.2%], and *N* = 290 [92.9%], respectively); overall, 89.4% (555/621) of participants completed the study. Demographic and clinical characteristics remained balanced at week 24 (Table 1).

**Table 1:** Participant demographic and disease characteristics at baseline and disease characteristics at week 24 (TP2 [FAS])

**Baseline demographics and disease characteristics**	**TCZ** **(** ***N*** **= 155)**	**TCZ→BAT1806/BIIB800** **(** ***N*** **= 154)**	**BAT1806/BIIB800** **(** ***N*** **= 312)**
Age, years	50.7 (12.4)	49.4 (11.7)	50.9 (11.9)
Female, *n* (%)	135 (87.1)	130 (84.4)	269 (86.2)
Weight, kg	67.4 (14.3)	65.9 (15.5)	66.2 (13.6)
Anti-CCP positive, *n* (%)	129 (83.2)	121 (78.6)	259 (83.0)
RF positive, *n* (%)	114 (73.5)	112 (72.7)	244 (78.2)
Previous b/tsDMARD use, *n* (%)	53 (34.2)	57 (37.0)	99 (31.7)
TJC68	23.9 (12.2)	23.6 (11.7)	22.5 (12.3)
SJC66	15.2 (8.1)	15.1 (7.1)	14.1 (7.8)
DAS28-CRP	5.89 (0.85)	5.89 (0.84)	5.81 (0.94)
DAS28-ESR	6.71 (0.95)	6.73 (0.83)	6.64 (0.88)
CRP, mg/L	19.7 (25.9)	18.1 (23.8)	18.9 (22.9)
ESR, mm/h	50.7 (22.0)	48.8 (25.2)	48.4 (22.5)
HAQ-DI	1.6 (0.5)	1.6 (0.6)	1.6 (0.6)
Pain VAS, mm	66.9 (18.7)	65.4 (20.3)	66.5 (19.5)
Participant GA VAS (0–100), mm	70.5 (15.6)	69.8 (18.7)	70.1 (17.5)
Physician GA VAS (0–100), mm	70.3 (13.4)	70.6 (16.3)	67.8 (14.8)
**Disease characteristics at week 24**	**TCZ** **(*N*** **= 145)**	**TCZ**→**BAT1806/BIIB800** **(*N*** **= 142)**	**BAT1806/BIIB800** **(*N*** **= 290)**
DAS28-CRP	3.37 (1.11)	3.31 (1.21)	3.18 (1.06)
DAS28-ESR	3.65 (1.34)	3.52 (1.54)	3.32 (1.50)
CRP, mg/L	1.96 (6.66)	2.70 (8.03)	1.79 (7.55)
ESR, mm/h	13.4 (14.3)	12.4 (12.6)	11.4 (10.7)
HAQ-DI	0.95 (0.62)	0.93 (0.58)	0.96 (0.58)
Pain VAS, mm	38.1 (20.8)	36.0 (20.1)	37.1 (20.5)
Participant GA VAS (0–100), mm	41.0 (20.9)	37.8 (20.1)	40.1 (20.7)
Physician GA VAS (0–100), mm	31.1 (18.9)	29.9 (20.1)	28.6 (17.8)

Data are mean (SD) unless stated otherwise

*bDMARD* Biological disease-modifying antirheumatic drug, *CCP* Cyclic citrullinated peptide, *CRP* C-reactive protein, *DAS28* Disease activity score on 28 joints, *ESR* Erythrocyte sedimentation rate, *FAS* Full analysis set, *GA* Global assessment, *HAQ-DI* Health assessment questionnaire – Disability index, *RF* Rheumatoid factor, *SD* Standard deviation, *SJC66* Swollen joint count of 66 joints, *TCZ* Tocilizumab reference product, *TJC68* Tender joint count of 68 joints, *TP2* Treatment period 2, *tsDMARD* Targeted synthetic disease-modifying antirheumatic drug, *VAS* Visual analogue scale

The treatment groups were similar prior to the start of TP2 also in terms of intercurrent events (type and occurrence percentage) (Supplemental Fig. 1) [5, 6].

**Fig. 2:**
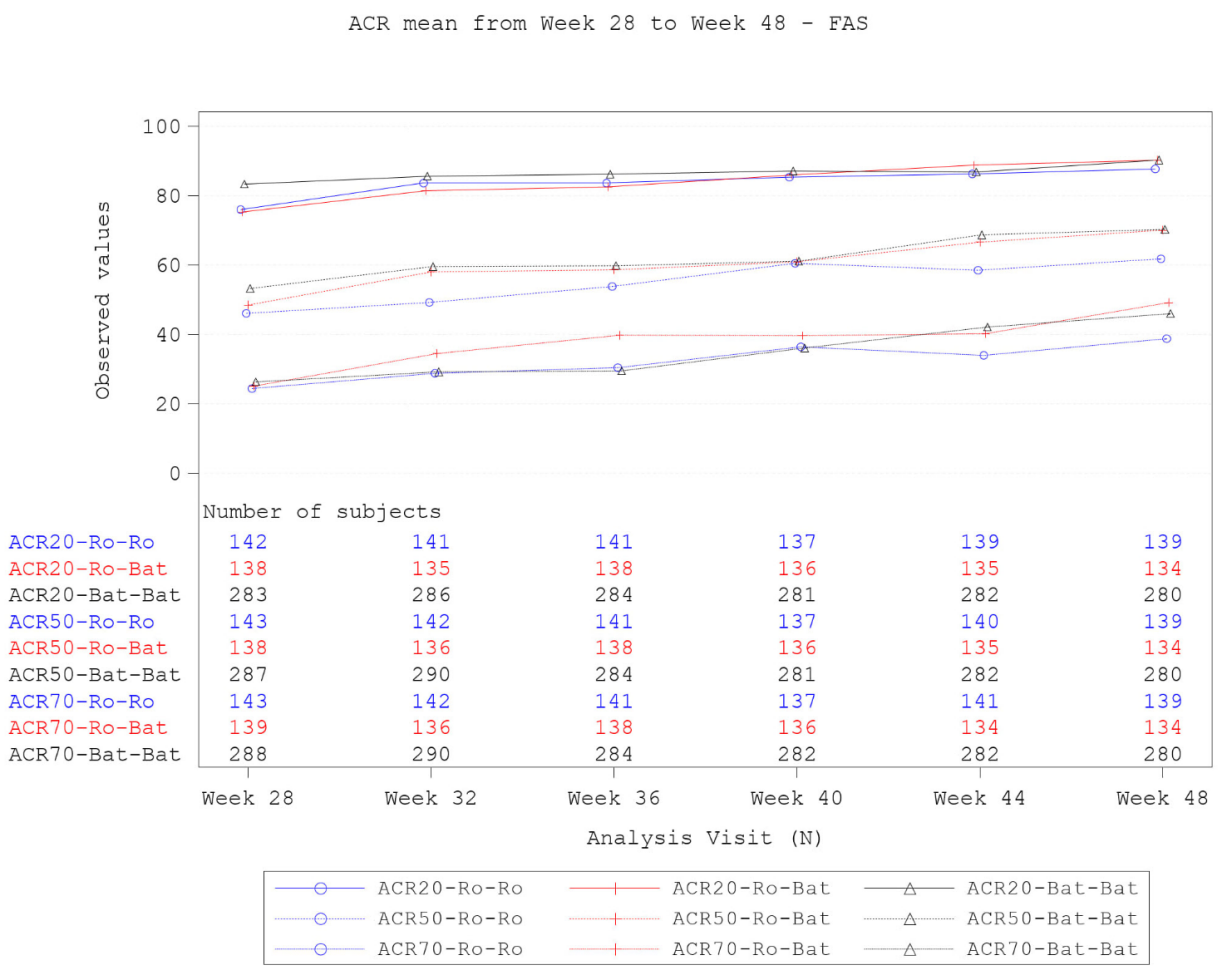
Proportions of participants achieving ACR20, ACR50, and ACR70 during TP2 (FAS). Participants were considered as entered into TP2 if the last study drug administration visit was on or after week 24, or participants received the first dose of BAT1806/BIIB800 for the switched group (TCZ→BAT1806/BIIB800). ACR response rates were calculated based on the number of participants with evaluable data at each time point, with data as observed; participants with missing data were considered as nonresponders. FAS included all randomized participants. *ACR20/50/70* ≥ 20%/50%/70% response in the American College of Rheumatology criteria, *FAS* Full analysis set, *TCZ* Tocilizumab reference product, *TP2* Treatment period 2

Proportions of ACR20, ACR50, and ACR70 responders were similar between treatment groups throughout TP2 (Fig. 2). At week 48, the proportions of participants who achieved ACR20 in the TCZ, TCZ→BAT1806/BIIB800, and BAT1806/BIIB800 groups were 87.8% (122/139), 90.3% (121/134), and 90.4% (253/280), respectively. The proportions of participants who achieved ACR50 at week 48 were 61.9% (86/139), 70.1% (94/134), and 70.4% (197/280), respectively, and for ACR70 were 38.8% (54/139), 49.3% (66/134), and 46.1% (129/280), respectively.

**Fig. 3:**
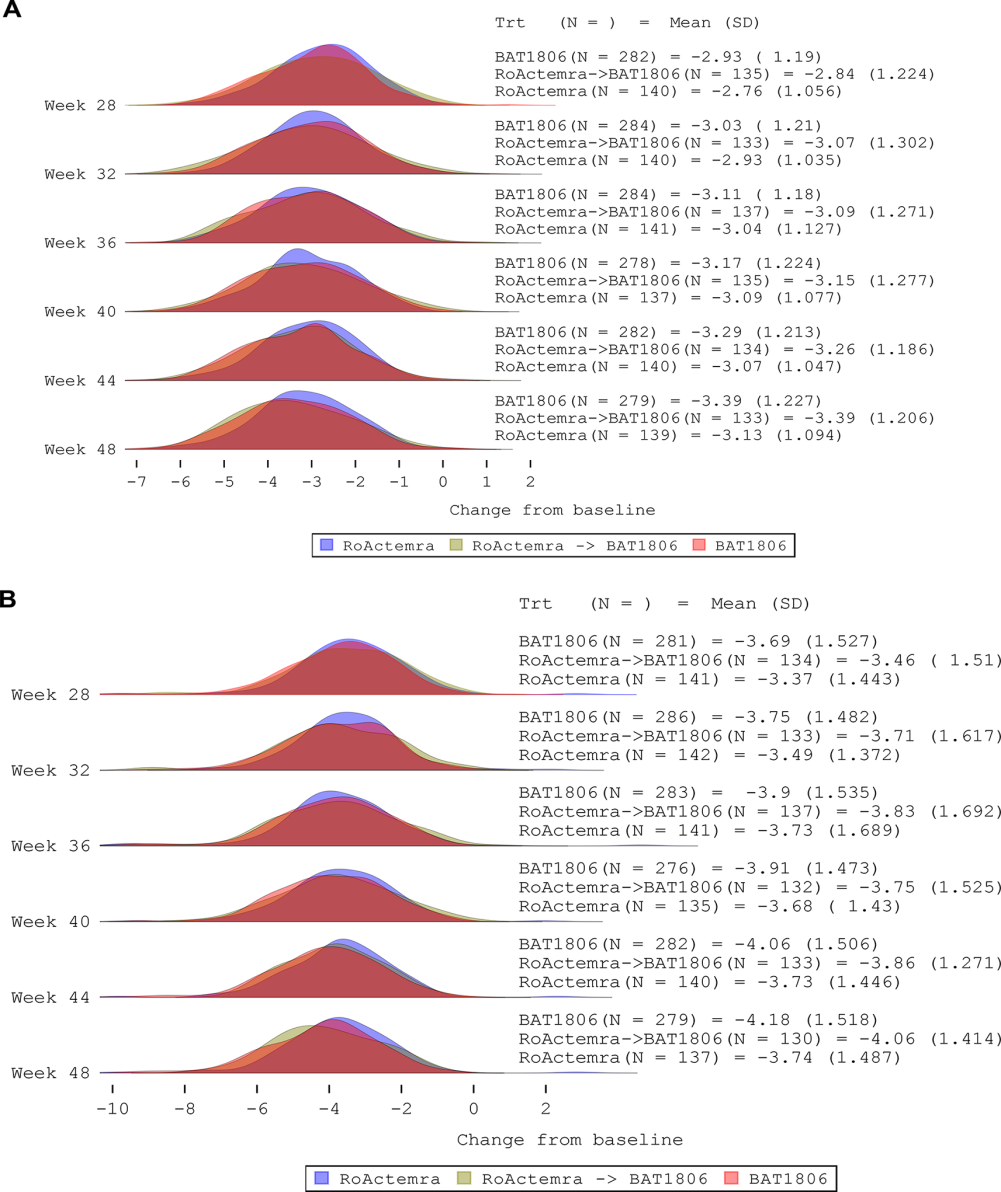
DAS28 (CRP [**A**] and ESR [**B**]) for the treatment groups by TP2 visits (FAS). *CRP* C-reactive protein, *DAS28* Disease Activity Score on 28 joints, *ESR* Erythrocyte sedimentation rate, *FAS* Full analysis set, *SD* Standard deviation, *TCZ* Tocilizumab reference product, *TP2* Treatment period 2

DAS28 (ESR and CRP) responses at each time point in TP2 were similar across the three treatment groups (Fig. 3, Supplemental Table 1).

The treatment groups displayed similar longitudinal trajectories in terms of DAS28-ESR and DAS28-CRP responses (Supplemental Fig. 2A–D). From the MMRM analyses, no differences between treatment arms with regards to DAS28-ESR and DAS28-CRP were observed, with overlapping 95% CIs at all time points. At week 48, mean (standard deviation [SD]) changes in DAS28-ESR were − 3.7 (1.5), − 4.1 (1.4), and − 4.2 (1.5) in the TCZ, TCZ→BAT1806/BIIB800, and BAT1806/BIIB800 groups, respectively. At week 48, mean (SD) changes in DAS28-CRP were − 3.1 (1.1), − 3.4 (1.2), and − 3.4 (1.2), respectively.

In the PK analysis, C_trough_ geometric mean values for the TCZ, TCZ→BAT1806/BIIB800, and BAT1806/BIIB800 groups were 12.4 μg/mL (coefficient of variation [CV]% 180.7), 12.7 μg/mL (CV% 157.0), and 13.1 µg/mL (CV% 126.5), respectively, at week 28, and 13.5 µg/mL (CV% 121.0), 13.4 µg/mL (CV% 125.1), and 12.3 µg/mL (CV% 150.6), respectively, at week 44.

In TP2, 345 (59.8%) participants experienced a total of 1179 AEs, including 323 events in 91 (62.8%), 287 events in 92 (64.8%), and 569 events in 162 (55.9%) participants in the TCZ, TCZ→BAT1806/BIIB800, and BAT1806/BIIB800 groups, respectively (Table 2). Overall, 344 (59.6%) participants reported 1163 TEAEs; 318 TEAEs in 90 (62.1%) participants in the TCZ group, 286 TEAEs in 92 (64.8%) participants in the TCZ→BAT1806/BIIB800 group, and 559 TEAEs in 162 (55.9%) participants in the BAT1806/BIIB800 group. The most common TEAEs in TP2 across treatment groups were upper respiratory tract infection, reported by 41 of 577 (7.1%) participants, and leukopenia, reported in 40 of 577 (6.9%) participants. Related TEAEs occurred in 59 (40.7%), 64 (45.1%), and 112 (38.6%) participants in the TCZ, TCZ→BAT1806/BIIB800, and BAT1806/BIIB800 groups, respectively. Most were mild in severity (mild: 56 [38.6%] participants, 56 [39.4%] participants, and 101 [34.8%] participants, respectively; moderate: 14 [9.7%] participants, 18 [12.7%] participants, and 26 [9.0%] participants, respectively; severe: 2 [1.4%] participants, 0 [0%] participants, and 1 [0.3%] participants, respectively). Five serious TEAEs were reported in four (2.8%) participants in the TCZ group, five serious TEAEs in five (3.5%) participants in the TCZ→BAT1806/BIIB800 group, and nine serious TEAEs in eight (2.8%) participants in the BAT1806/BIIB800 group.

**Table 2:** Summary of TEAEs by preferred term during TP2 and up to week 52 (safety set)

Participants with TEAE, *n* (%)	TCZ (*N* = 145)	TCZ→BAT1806/BIIB800 (*N* = 142)	BAT1806/BIIB800 (*N* = 290)
TEAE	90 (62.1)	92 (64.8)	162 (55.9)
Upper respiratory tract infection	15 (10.3)	6 (4.2)	20 (6.9)
Leukopenia	15 (10.3)	10 (7.0)	15 (5.2)
ALT increased	7 (4.8)	15 (10.6)	13 (4.5)
Hyperlipidemia	6 (4.1)	7 (4.9)	21 (7.2)
Hepatic function abnormal	9 (6.2)	10 (7.0)	10 (3.4)
Neutropenia	7 (4.8)	9 (6.3)	12 (4.1)
Anemia	4 (2.8)	8 (5.6)	5 (1.7)
Related TEAE	59 (40.7)	64 (45.1)	112 (38.6)
Leukopenia	15 (10.3)	9 (6.3)	12 (4.1)
ALT increased	6 (4.1)	13 (9.2)	11 (3.8)
Upper respiratory tract infection	9 (6.2)	5 (3.5)	14 (4.8)
Neutropenia	7 (4.8)	9 (6.3)	12 (4.1)
Hepatic function abnormal	9 (6.2)	10 (7.0)	9 (3.1)
Serious TEAE^a^	4 (2.8)	5 (3.5)	8 (2.8)
Related serious TEAE^a^	1 (0.7)	1 (0.7)	2 (0.7)
Fatal TEAEs	0	0	0

^a^No serious TEAE occurred in more than two participants (≥ 1.5%) in any treatment group. Only TEAEs that occurred in ≥ 5% of participants are listed

Safety set included all randomized participants who had received ≥ 1 dose of study drug

N represents the number of participants who received study treatment in TP2

*ALT* Alanine aminotransferase, *TCZ* Tocilizumab reference product, *TEAE* Treatment-emergent adverse event, *TP2* Treatment period 2

The only serious TEAE to occur in more than one participant in any one treatment group was tooth abscess, which occurred in two participants in each of the treatment groups. Of the serious TEAEs, one (pneumonia) in the TCZ group, one (laryngitis) in the TCZ→BAT1806/BIIB800 group, and two (pneumonia and salpingo-oophoritis) in the BAT1806/BIIB800 group were considered by the investigator to be possibly related to study treatment (Table 3). There were no fatal events in TP2.

**Table 3:** Summary of serious TEAEs by preferred term during TP2 and to week 52 (safety set)

Number of participants with serious TEAE (%)	TCZ (*N* = 145)	TCZ→BAT1806/BIIB800 (*N* = 142)	BAT1806/BIIB800 (*N* = 290)
Any serious TEAE	4 (2.8)	5 (3.5)	8 (2.8)
Pneumonia	1 (0.7)	0	1 (0.3)
Appendicitis	1 (0.7)	0	0
COVID-19	0	1 (0.7)	0
Laryngitis	0	1 (0.7)	0
Salpingo-oophoritis	0	0	1 (0.3)
Tooth abscess	2 (1.4)	2 (1.4)	2 (0.7)
Contusion	1 (0.7)	0	0
Joint injury	1 (0.7)	0	0
Patella fracture	1 (0.7)	0	0
Rib fracture	0	0	1 (0.3)
Syncope	0	1 (0.7)	0
Transient ischemic attack	0	0	1 (0.3)
Lumbar spinal stenosis	0	1 (0.7)	1 (0.3)
Pathological fracture	0	0	1 (0.3)
Spondylolisthesis	0	0	1 (0.3)
Acute myocardial infarction	0	0	1 (0.3)
Myocardial ischemia	0	0	1 (0.3)
Renal hamartoma	0	1 (0.7)	0

Safety set included all randomized participants who had received ≥ 1 dose of study drug

N represents the number of participants who received study treatment in TP2

*TCZ* Tocilizumab reference product, *TEAE* Treatment-emergent adverse event,

*TP2* Treatment period 2

**Fig. 4:**
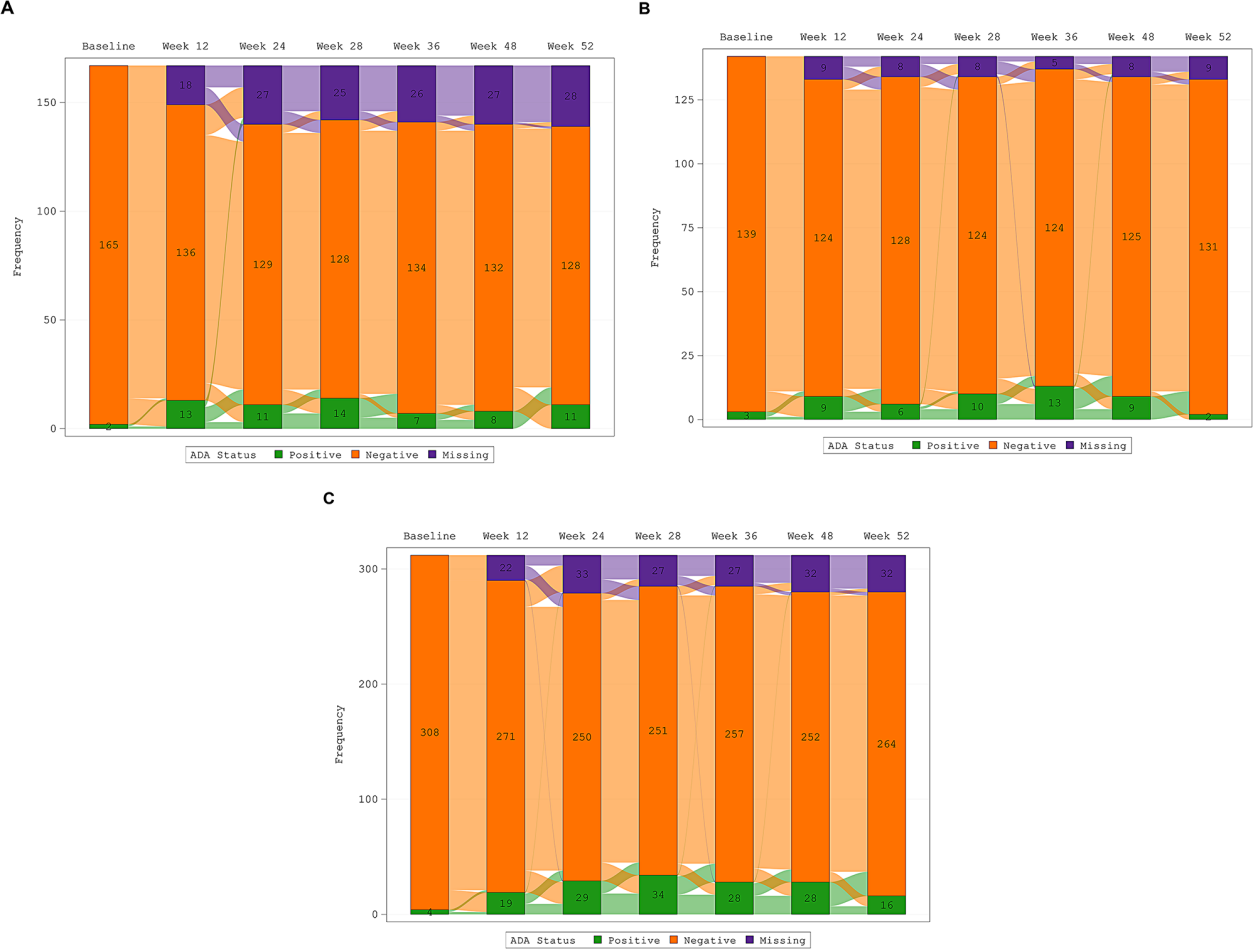
Sankey plot of ADA status at each visit during TP2: for (**A**) the TCZ group, (**B**) the TCZ→BAT1806/BIIB800 group, and (**C**) the BAT1806/BIIB800 group. *ADA* Antidrug antibody, *TCZ* Tocilizumab reference product, *TP2* Treatment period 2

In TP2, 18.6% (27/145), 16.2% (23/142), and 21.0% (61/290) of participants in the TCZ, TCZ→BAT1806/BIIB800, and BAT1806/BIIB800 groups, respectively, had ≥ 1 positive ADA result at any time point. Across the treatment groups, the majority of ADA-positive participants reported titers of < 20 or 20. All ADA-positive tests were also positive for nAbs, with the exception of a single result in the TCZ group. The point prevalence for participants testing positive for ADAs remained stable in all treatment groups, and the majority of positive cases transitioned from positive to negative across the study period (Fig. 4). At week 52 (8 weeks after the last dose of study drug), 11 (7.9%), 2 (1.5%), and 16 (5.7%) participants were ADA-positive in the TCZ, TCZ→BAT1806/BIIB800, and BAT1806/BIIB800 groups, respectively; the majority of these participants were also positive for nAbs at this time point (10 [7.2%], 2 [1.5%], and 15 [5.4%], respectively).

## Discussion

In TP2 BAT1806/BIIB800 showed comparable efficacy, safety, immunogenicity, and PK to TCZ, in a population of patients with RA and an inadequate response to MTX. These longer-term findings reinforce the results reported for TP1 and further support biosimilarity. The results do not suggest any detriment in terms of efficacy, safety, or immunogenicity following a switch from TCZ to BAT1806/BIIB800.

Efficacy as assessed by ACR response or change in DAS28 at 1 year was comparable across the treatment groups. No loss of efficacy was observed during TP2 in any treatment group [5], and was in line with reported efficacy at 1 year in published studies of the reference product [7, 8]. The safety profile of BAT1806/BIIB800 was comparable to that of TCZ up to week 52 and no new safety signals were identified.

Demonstrating PK similarity of a reference product and its biosimilar is a key aspect of a successful biosimilarity exercise [9]. No differences in PK parameters were observed between the treatment groups, with a similarly large magnitude of variability observed. These findings are consistent with results from the phase 1 PK study [4] and from TP1 of the present study [5]. Additionally, previous studies have reported that > 95% of the sIL-6R is bound at C_trough_ levels of 1 µg/mL [10] and that trough concentrations above 1 µg/mL could normalize CRP, whereas lower trough levels were associated with reduced DAS28 responses [11].

While the prevalence of ADAs in TP2 was comparable between the TCZ, TCZ→BAT1806/BIIB800, and BAT1806/BIIB800 groups, the overall prevalence was higher than historical values reported for the reference product in a pooled analysis of clinical studies (1.2%) [12]. This is likely to be due to the use of more sensitive and drug-tolerant ADA assays than those used previously [13]. Most ADA-positive participants had a titer of < 20 or 20, and transitioned between positive and negative status throughout the study, suggesting that the majority of the antibody responses were transient and not clinically meaningful. This is in line with the aforementioned pooled analysis of the reference product, which reported that for the majority of positive ADA results titers were low and ADAs were transient; that analysis also reported no impact of ADA positivity on efficacy [12]. Furthermore, the pooled analysis found no correlation between the presence of ADAs and either safety or PK events (including anaphylaxis, hypersensitivity, and injection-site reactions) [12].

The findings of this study must be considered in the context of several potential limitations. A single indication (RA) was chosen for the study population; this was selected as being the most sensitive population eligible for TCZ treatment. Based on the principle of extrapolation of indications, a demonstration of clinical similarity in one sensitive indication can be extrapolated to all labeled indications of the reference product, without the need for additional clinical studies, if adequately justified [12]. The study population was predominantly female; however, global prevalence data show that 70% of the population diagnosed with RA are women, [14] indicating that the study sample is broadly representative. The geographic locations of the study sites included China and four European countries, with limited enrollment of different ethnicities; as there is no evidence that ethnicity or race affects the PK of the reference product this is not considered likely to lessen the validity of the study results [15, 16]. Disease progression was not measured radiographically, however the clinical endpoints used in this study (ACR, DAS28) were agreed with regulatory agencies during the study design stage as being appropriately sensitive to detect differences between the biosimilar and reference product, should these exist. This study was designed to assess the possible impact of a single treatment switch from TCZ to BAT1806/BIIB800 after week 24. In clinical practice, multiple treatment switches may occur; post-approval studies may provide additional data about the use of biosimilars in real-world settings.

## Conclusion

In conclusion, the efficacy, safety, immunogenicity, and PK profiles in TP2 were comparable between the TCZ, TCZ→BAT1806/BIIB800, and BAT1806/BIIB800 treatment groups.

## Electronic supplementary material

Below is the link to the electronic supplementary material.

Supplementary Material 1

## Data Availability

The data that support the findings of this study are available from the authors on request. Patient-level data will be anonymized, and study documents will be redacted to protect the privacy of trial participants. Further information on data sharing is available via Biogen’s clinical transparency and data sharing policy.

## Abbreviations

ACR: American College of Rheumatology
ADA: Antidrug antibody
AE: Adverse event
ALT: Alanine aminotransferase
bDMARD: Biological disease-modifying antirheumatic drug
BOCF: Baseline observation carried forward
CCP: Cyclic citrullinated peptide
CRP: C-reactive protein
C_trough_: Serum trough concentration
CV: Coefficient of variation
DAS28: Disease Activity Score on 28 joints
DMARD: Disease-modifying antirheumatic drug
EOS: End of study
ESR: Erythrocyte sedimentation rate
FAS: Full analysis set
GA: Global Assessment
HAQ-DI: Health Assessment Questionnaire – Disability Index
IL-6R: Interleukin-6 receptor
MMRM: Mixed model for repeated measures
MTX: Methotrexate
nAb: Neutralizing antibody
PK: Pharmacokinetic
RA: Rheumatoid arthritis
RF: Rheumatoid factor
SD: Standard deviation
SJC68: Swollen joint count of 68 joints
TB: Tuberculosis
TCZ: Tocilizumab reference product
TEAE: Treatment-emergent adverse event
TP: Treatment period
tsDMARD: Targeted synthetic disease-modifying antirheumatic drug
VAS: Visual analogue scale
