# Supplementary material for: A phase 3, randomized, double-blind, active-controlled clinical trial to compare BAT1806/BIIB800, a tocilizumab biosimilar, with tocilizumab reference product in participants with moderate-to-severe rheumatoid arthritis with inadequate response to methotrexate: treatment period 2 analysis (week 24 to week 48)
Source: Arthritis Res Ther. 2024 Sep 7;26:157. doi: 10.1186/s13075-024-03375-w (PMC11380339; doi:10.1186/s13075-024-03375-w)
Supplement: Supplementary file 1 — Supplementary Material 1 [file 13075_2024_3375_MOESM1_ESM.docx]

# SupplementaL Material

**Supplemental Table 1** DAS28-CRP and DAS28-ESR: Change from baseline and remission frequencies post week 24 through week 48 (FAS)

| **Visit** | **Statistic** | **DAS28-CRP** | | | **DAS28-ESR** | | |
| --- | --- | --- | --- | --- | --- | --- | --- |
|  |  | **TCZ (*N* = 155)** | **TCZ****🡪BAT1806 (*N* = 154)** | **BAT1806 (*N* = 312)** | **TCZ (*N* = 155)** | **TCZ🡪BAT1806 (*N* = 154)** | **BAT1806 (*N* = 312)** |
| Week 28 | *n* | 140 | 135 | 282 | 141 | 134 | 281 |
|  | Mean (SD) | –2.760 (1.0562) | –2.838 (1.2236) | –2.929 (1.1900) | –3.365 (1.4427) | –3.462 (1.5100) | –3.686 (1.5270) |
|  | EULAR remission, *n* (%)^a^ | 47 (30.3) | 50 (32.5) | 123 (39.4) | 39 (25.2) | 43 (27.9) | 99 (31.7) |
| Week 32 | *n* | 140 | 133 | 284 | 142 | 133 | 286 |
|  | Mean (SD) | –2.929 (1.0345) | –3.070 (1.3016) | –3.028 (1.2096) | –3.492 (1.3720) | –3.708 (1.6171) | –3.753 (1.4822) |
|  | EULAR remission, *n* (%)^a^ | 54 (34.8) | 60 (39.0) | 125 (40.1) | 44 (28.4) | 52 (33.8) | 121 (38.8) |
| Week 36 | *n* | 141 | 137 | 284 | 141 | 137 | 283 |
|  | Mean (SD) | –3.041 (1.1267) | –3.091 (1.2710) | –3.113 (1.1805) | –3.728 (1.6895) | –3.829 (1.6922) | –3.896 (1.5346) |
|  | EULAR remission, *n* (%)^a^ | 64 (41.3) | 58 (37.7) | 141 (45.2) | 50 (32.3) | 57 (37.0) | 123 (39.4) |
| Week 40 | *n* | 137 | 135 | 278 | 135 | 132 | 276 |
|  | Mean (SD) | –3.085 (1.0773) | –3.154 (1.2766) | –3.174 (1.2239) | –3.680 (1.4298) | –3.754 (1.5245) | –3.915 (1.4726) |
|  | EULAR remission, *n* (%)^a^ | 62 (40.0) | 65 (42.2) | 144 (46.2) | 51 (32.9) | 55 (35.7) | 119 (38.1) |
| Week 44 | *n* | 140 | 134 | 282 | 140 | 133 | 282 |
|  | Mean (SD) | –3.070 (1.0468) | –3.259 (1.1856) | –3.285 (1.2126) | –3.729 (1.4462) | –3.858 (1.2710) | –4.062 (1.5063) |
|  | EULAR remission, *n* (%)^a^ | 63 (40.6) | 71 (46.1) | 158 (50.6) | 54 (34.8) | 56 (36.4) | 137 (43.9) |
| Week 48 | *n* | 139 | 133 | 279 | 137 | 130 | 279 |
|  | Mean (SD) | –3.128 (1.0936) | –3.394 (1.2062) | –3.388 (1.2273) | –3.738 (1.4868) | –4.061 (1.4139) | –4.183 (1.5182) |
|  | EULAR remission, *n* (%)^a^ | 68 (43.9) | 80 (51.9) | 174 (55.8) | 56 (36.1) | 67 (43.5) | 151 (48.4) |

^a^ Percentages calculated using *N* as the denominator

*CRP* C-reactive protein, *DAS28* Disease Activity Score on 28 joints, *ESR* Erythrocyte sedimentation rate, *EULAR* European Alliance of Associations for Rheumatology, *FAS* Full analysis set, *SD* Standard deviation, *TCZ* Tocilizumab reference product

**Supplemental Fig. 1** Proportion of participants completing follow-up post intercurrent event occurred at either week 12 or week 24 (FAS) for **A** the TCZ group, **B** the TCZ**🡪**BAT1806/BIIB800 group, and **C** the BAT1806/BIIB800 group

**A**

participant

**B**

participant

**C**

participant

*ACR* American College of Rheumatology, *FAS* Full analysis set, *ICE* Intercurrent event,
*TCZ* Tocilizumab reference product, *TP2* Treatment period 2

**Supplemental Fig. 2** Least squares mean difference (95% CI) over scheduled visits in
**A** DAS28-ESR and **B** DAS28-CRP during TP2 (FAS) as observed, and **C** DAS28-ESR and
**D** DAS28-CRP with BOCF imputation

**A** (as observed)

**B** (as observed)

**C** (with BOCF imputation)

**D** (with BOCF imputation)

^a^ 95% CI values were based on mixed-effect model for repeated measures with treatment arm, visit, randomization stratification factors (region and previous biological or targeted synthetic DMARD use), baseline result, and treatment-by-visit interactions in the model. Percentages are based on the number of participants in the FAS and missing data are not imputed

*BOCF* Best observation carried forward, *CI* Confidence interval, *CRP* C-reactive protein,
*DAS28* Disease Activity Score on 28 joints, *DMARD* Disease-modifying antirheumatic drug,
*ESR* Erythrocyte sedimentation rate, *FAS* Full analysis set, *LSM* Least squares mean,
*TCZ* Tocilizumab reference product, *TP2* Treatment period 2
